# Case Report: Mitochondrial insufficiency underlies cholinergic failure in post-vaccination long COVID: a candidate treatment protocol

**DOI:** 10.3389/fmed.2026.1813032

**Published:** 2026-07-21

**Authors:** Tom Goulding

**Affiliations:** Northeastern University, Boston, MA, United States

**Keywords:** cholinergic deficiency, citric acid, mitochondrial dysfunction, recovery protocol, symptom suppression

## Abstract

An 80-year-old male with severe long COVID developed acute deterioration following COVID-19 vaccination despite ongoing cholinergic therapy (Mestinon 90 mg daily). A comprehensive mitochondrial support protocol targeting NAD+ repletion, electron transport chain function, and oxidative stress was initiated, with all components delivered in fresh-squeezed orange juice. Clinical response was dramatic within 48 h, with sustained functional recovery over 6 weeks. Challenge-rechallenge testing demonstrated rapid symptom recurrence and resolution with three key observations: missed doses, wrong component sequence, and substitution of water for orange juice. This final observation suggests citric acid may function as a rate-limiting Krebs cycle substrate, consistent with impaired endogenous citrate synthesis in severe long COVID. This case suggests that cholinergic support may require concurrent metabolic foundation including direct substrate supplementation, and some long COVID manifestations may be metabolically reversible with multi-pathway intervention. As a single self-case report, these findings generate hypotheses for systematic investigation rather than establishing treatment guidelines.

## Introduction

Long COVID affects over 70% of survivors beyond 4 months post-infection, with mitochondrial dysfunction emerging as a key pathophysiological mechanism (1, 2). Post-vaccination worsening of long COVID symptoms poses significant challenges in elderly patients with pre-existing post-viral syndrome (3, 4). Standard therapeutic approaches have shown limited efficacy, particularly in severe cases with multi-system involvement.

We present the case of an 80-year-old male professor with PhD-level training in biochemistry who developed dramatic deterioration following COVID-19 vaccination. The patient independently developed a comprehensive mitochondrial support protocol that produced rapid functional recovery. Notably, inadvertent challenge-rechallenge observations revealed that citric acid supplementation via fresh-squeezed orange juice was essential for protocol efficacy, suggesting substrate limitation as a critical but overlooked component of long COVID pathophysiology. This case has implications for accessible, low-cost interventions in post-viral metabolic dysfunction.

## Case presentation

An 80-year-old male professor, previously in excellent health with no chronic conditions and high baseline fitness, developed severe COVID-19 requiring hospitalization in December 2024. The acute infection necessitated 4 days of inpatient care followed by 4 days of inpatient rehabilitation. Post-hospitalization, he developed typical long COVID manifestations including profound fatigue, cognitive impairment, balance disturbances, and exercise intolerance.

Eight months post-hospitalization, the patient was prescribed Mestinon (pyridostigmine bromide) 90 mg daily for emerging balance problems. This therapy provided minimal benefit over 2 months, with persistent severe functional limitations. In early September 2025, 9 months post-infection, the patient received his fifth COVID-19 vaccination (Moderna) on September 5th. Within 24 h, he experienced immediate and severe clinical deterioration despite ongoing Mestinon therapy, including severe energy depletion, cognitive decline with memory impairment, severe balance instability with fall risk, profound weakness, sensation of spatial disorientation, and complete functional collapse requiring assistance with basic self-care. The severity necessitated medical leave from his professorship at Northeastern University in November 2025.

Based on his biochemistry training and emerging research linking long COVID to mitochondrial dysfunction (1, 2, 5), the patient developed a comprehensive evidence-based protocol in late November 2025. The protocol addressed multiple rate-limiting steps in cellular energy production: NAD+ repletion with nicotinamide riboside 500 mg daily (6–8); electron transport chain support with ubiquinol one gram daily, high-dose B-complex vitamins, zinc 30 mg daily, and copper two mg daily (9–11); ATP buffering with creatine monohydrate five grams daily (12); antioxidant protection with N-acetylcysteine 900 mg daily (13, 14); and continued cholinergic support with Mestinon 90 mg daily (15, 16). Enhanced vitamin B12 supplementation included two chewable tablets daily plus intramuscular injections twice monthly. The patient’s established antidepressant nortriptyline 20 mg daily was continued (17, 18).

All components were administered with seven ounces of fresh-squeezed orange juice three times daily, initially selected as a palatable delivery medium. Components were divided across morning, midday, and evening doses with 5 to 6 h intervals during waking hours and 12-plus hour intervals during sleep. Clinical response was dramatic within 48 h: significant energy restoration from bedridden to functional, marked reduction in cognitive impairment, resolution of balance disturbances, and restoration of normal activities including return to 1.5-mile walks and strength training.

The causal relationship between protocol and improvement was demonstrated through three inadvertent challenge-rechallenge observations. First, missed morning doses on two occasions resulted in symptom recurrence within 4–6 h, with resolution within 1–2 h after taking the delayed dose. Second, intentional reversal of component sequence for 5 days provided minimal benefit, demonstrating that timing sequence was mechanistically important. Specifically, component sequence reversal tested whether administering supplements in reverse temporal order altered efficacy, based on the hypothesis that timing of specific cofactors relative to orange juice administration might matter. Third, and most significantly, inadvertent substitution of water for orange juice while maintaining all other protocol components at correct doses resulted in complete loss of symptom control. Morning and noon doses administered with water left the patient bedridden. The evening dose administered with seven ounces of fresh-squeezed orange juice produced noticeable improvement within 30 min, ability to walk one-eighth mile at 45 min, and complete functional recovery within 1–2 h.

This final observation isolates the orange juice component, specifically its citric acid content of approximately 500–700 mg per serving, as an essential rate-limiting substrate for protocol efficacy. The rapid response to citrate repletion (30–45 min) is consistent with acute Krebs cycle substrate deficiency rather than cofactor limitation alone. Balance instability and spatial disorientation served as particularly sensitive early indicators of metabolic decompensation, appearing within hours of missed doses and resolving rapidly with dose administration.

Clinical improvements have been sustained for over 6 weeks as of January 2026 with continued protocol adherence. The patient maintains near-complete functional recovery and planned return to work. Subsequent protocol optimization has allowed reduction of medication burden while maintaining symptom control. Follow-up continues under medical supervision at Massachusetts General Hospital.

## Discussion

This case demonstrates several important observations with potential therapeutic implications for post-vaccination long COVID. The dramatic post-vaccination deterioration, occurring 9 months post-infection despite ongoing cholinergic therapy, represents a recognized but poorly understood phenomenon (3, 4, 19). The patient’s case suggests that COVID-19 vaccination can trigger acute exacerbation in patients with ongoing long COVID, emphasizing the need for careful risk-benefit assessment and potential timing strategies.

The most significant finding is that cholinergic support with Mestinon, ineffective in isolation for over 2 months, demonstrated dramatic efficacy when combined with comprehensive mitochondrial support. This suggests that cholinergic dysfunction in long COVID may be secondary to underlying metabolic impairment (1, 2, 15, 16). Acetylcholine signaling requires adequate cellular ATP, and isolated pathway interventions may fail where multi-targeted approaches succeed.

The inadvertent water substitution experiment provides compelling evidence that substrate provision is as critical as cofactor supplementation. Complete protocol failure with water despite all supplements and medications being present, followed by rapid recovery (30–45 min) with orange juice reintroduction, isolates citric acid as an essential component. In healthy individuals, citrate is synthesized endogenously via citrate synthase. This case suggests that severe long COVID impairs this synthetic capacity, creating substrate limitation that prevents Krebs cycle operation even when adequate cofactors are present (1, 2, 5).

By providing exogenous citrate (approximately 1.5 to two grams daily from orange juice), this impaired synthesis is bypassed, allowing the Krebs cycle to operate with the supplemented cofactors. The rapid response time is consistent with citrate absorption from small intestine (15–30 min), reaching mitochondria via bloodstream (20–40 min), with immediate Krebs cycle resumption upon substrate provision. This represents a fundamental reframing from cofactor deficiency to substrate plus cofactor deficiency as the pathophysiology of metabolic long COVID.

The 48-h response to initial protocol initiation, and even more rapid 30–45 min response to citrate repletion, suggests acute substrate and cofactor deficiency rather than structural mitochondrial damage (1, 2, 9, 20). If mitochondrial damage were primarily structural, recovery would require weeks to months for mitochondrial biogenesis. The rapid response suggests that providing adequate substrate and cofactors allows existing but metabolically starved mitochondria to resume function. This finding is clinically optimistic, suggesting some long COVID manifestations are metabolically reversible with appropriate intervention rather than representing permanent tissue damage.

Simple, inexpensive citrate supplementation (approximately three dollars daily via fresh orange juice, or less with citrate supplements) may benefit patients with metabolic long COVID symptoms. The rapid response (30–45 min) enables straightforward clinical testing. While fresh-squeezed orange juice was used in this case and provides additional beneficial components including vitamin C, flavonoids, and potassium, citrate is available from multiple sources including potassium citrate, sodium citrate, and calcium citrate supplements. The optimal formulation and whether synergistic components in orange juice are necessary for full efficacy remain to be determined.

Balance instability as the earliest and most reliable indicator of protocol failure is mechanistically significant. The cerebellum has the highest mitochondrial density in the brain, and vestibular-cerebellar integration requires continuous high ATP availability. Balance impairment as the first system to fail with substrate depletion, and first to recover with substrate provision, supports the hypothesis that neurological manifestations of long COVID are primarily energetic rather than structural, and thus potentially reversible. The water substitution experiment isolates orange juice as an essential protocol component. While citric acid is proposed as the primary active constituent based on its direct role as a Krebs cycle substrate and the rapid response timeline consistent with citrate absorption kinetics, the possible contributions of vitamin C, flavonoids, potassium, and carbohydrates in orange juice cannot be excluded from this single-case observation. Future investigation should include pure citrate supplementation as a direct comparator to isolate the active component.

This case has important limitations. As a single-patient self-experiment with no blinding or randomization, generalizability is uncertain. The non-blinded, self-administered nature of all observations means placebo and nocebo effects cannot be excluded. The patient’s PhD-level biochemistry training enabled protocol design that may not be reproducible by typical patients. No mechanistic laboratory investigations were performed; the citric acid hypothesis requires biochemical validation in future studies using isotope tracing or *in vitro* models. The protocol involves multiple simultaneous interventions, making individual component contributions difficult to isolate beyond the water-substitution observation. Validated patient-reported outcome instruments (SF-36, CPET, cognitive assessments) were not administered; functional recovery descriptions represent unvalidated clinical observations. However, the robust challenge-rechallenge observations, particularly the water substitution experiment, provide strong evidence for causal relationships rather than spontaneous recovery or placebo effect.

## Conclusion

The following observations are derived from a single self-case report and are intended to generate hypotheses for systematic investigation rather than establish treatment guidelines. This case suggests that post-vaccination worsening of long COVID can occur months after initial infection, and that cholinergic support may require concurrent metabolic foundation to be effective. Most significantly, citric acid supplementation appears essential for protocol efficacy, suggesting Krebs cycle substrate limitation as a key but overlooked pathophysiological mechanism in severe long COVID. The rapid response to substrate provision (30–45 min), combined with multi-pathway cofactor support, suggests that some long COVID manifestations are metabolically reversible rather than representing permanent tissue damage. The accessibility and low cost of citric acid supplementation (via orange juice or supplements) warrants systematic investigation in long COVID populations. Balance instability may serve as a sensitive biomarker for metabolic decompensation. This substrate plus cofactor model represents a potentially important reframing of long COVID pathophysiology with implications for treatment development.

## Data Availability

All relevant data is contained within the article.
